# Screening, diagnosis and treatment of hypertension in obese children: an international policy comparison

**DOI:** 10.1007/s40620-016-0277-6

**Published:** 2016-03-03

**Authors:** Aleid J. G. Wirix, Jelle Verheul, Jaap W. Groothoff, Jeroen Nauta, Mai J. M. Chinapaw, Joana E. Kist-van Holthe

**Affiliations:** 1 Department of Public and Occupational Health, EMGO Institute for Health and Care Research, VU University Medical Center, van der Boechorststraat 7, 1081BT Amsterdam, The Netherlands; 2 Department of Paediatric Nephrology, Emma Children’s Hospital/Academic Medical Center, Amsterdam, The Netherlands; 3 Department of Paediatric Nephrology, Erasmus Medical Center, Rotterdam, The Netherlands

**Keywords:** Hypertension, Obesity, Pediatric nephrology

## Abstract

Hypertension in obese children may require a different diagnostic and treatment approach from that for children with secondary hypertension, yet there is neither consensus nor a clear guideline. The aim of this study was to assess how obese children with hypertension are currently diagnosed and treated by paediatric nephrologists, what obstacles exist and what can be improved. In the period May–November 2014, an online questionnaire was sent to all members of the European Society for Paediatric Nephrology (n = 2148). Questions focused on current practices and obstacles regarding screening, diagnosis and treatment of hypertension in obese children. A total of 214 paediatric nephrologists responded. Although nearly 100 % agreed that screening of obese children for hypertension is indicated, it was current practice in only 56 % of participating countries; 88 % of respondents diagnosed hypertension with 24-h ambulatory blood pressure measurement. Diagnostics used to rule out causes or consequences of hypertension varied among the respondents; they included, in particular, the use of serum renin/aldosterone, urine sodium/potassium, and dimercaptosuccinic acid scan. Concerning treatment, 45 % of respondents preferred to start treatment with a lifestyle program, 2 % with antihypertensive medication, and 40 % with both. For 73 % of respondents, angiotensin-converting enzyme-inhibitors or angiotensin receptor blockers were the drugs of first choice. The findings of this study emphasize the urgent need for an international guideline for screening, diagnosis and treatment of hypertension in obese children.

**Electronic supplementary material:**

The online version of this article (doi:10.1007/s40620-016-0277-6) contains supplementary material, which is available to authorized users.

## Introduction

Overweight and obesity in children continues to be an increasing public health problem. As overweight and obesity are important risk factors for elevated blood pressure, hypertension is increasingly diagnosed in children as well. The prevalence of hypertension in (non-selected) schoolchildren aged 3–18 years of normal weight is 3–5 %, with overweight 4–14 %, and in obese children 11–33 % [1–6]. If not identified and treated early, hypertension can lead to atherosclerosis, cardiovascular disease and renal failure, and impose an important burden of disease [7].

The (US) National High Blood Pressure Education Program (NHBPEP) Working group on High Blood Pressure in Children and Adolescents (Fourth Report) as well as the European Society of Hypertension have provided guidelines for the diagnosis and treatment of hypertension [8, 9]. However, hypertension in obese children may need a different diagnostic and treatment approach from that for children with secondary hypertension. Obesity-related hypertension, often referred to as primary hypertension, is often less severe and less symptomatic in comparison to secondary hypertension [10]; thus, for example, a consultation with an ophthalmologist to check for hypertensive retinopathy might not be necessary. In addition, a lifestyle intervention might suffice as treatment for hypertension in obesity, whereas secondary hypertension most likely requires pharmacological treatment [11]. However, there is neither consensus nor a clear guideline regarding the screening, diagnosis and treatment of obese children with hypertension.

The aim of this study was to assess how obese children with hypertension are currently diagnosed and treated by paediatric nephrologists, and to explore possible obstacles to their management and what should be improved.

## Methods

Current practice of screening, diagnosis and treatment of hypertension in obese children was investigated through an online questionnaire (SurveyMonkey^®^, Palo Alto, CA, USA). The questionnaire (including up to two reminders) was sent to all members of the European Society for Paediatric Nephrology (n = 2148) in the period May–November 2014. The questionnaire consisted of 18 questions: 16 closed- and two open-ended questions (see Supplementary information 1). The questions focused on current practices and obstacles regarding screening, diagnosis and treatment of hypertension in obese children and suggestions to improve these aspects. The closed-ended questions were analysed with Microsoft Excel 2010. Data are expressed as percentages of respondents. The statistical analyses were performed with SPSS software version 20.0 (SPSS Inc., Chicago, IL, USA). Differences in diagnostics and treatment of hypertension between Europe and Asia were tested with *X* ^2^ tests. For the analysis of the open-ended questions, the answers were first coded according to an open-coded technique, because of the explorative nature of the open questions. The codes were then categorized into themes, which resulted in a list of topics representing the most frequent answers. By combining the codes under overarching categories, a clear outline of the relevant information was provided.

## Results

A total of 214 paediatric nephrologists filled out the questionnaire. Of the respondents, 65 % worked in Europe, 25 % in Asia, 4 % in South America, 3 % in Oceania and 2 % in North America. For the number of respondents per country, see Supplementary information 2. Concerning their employment, 70 % (n = 164) worked at a university hospital, 18 % (n = 43) in a general hospital, 9 % (n = 21) in a private clinic, and 2 % (n = 5) in a paediatric hospital.

According to 97 % (203/209) of the respondents, all obese children should be screened for hypertension; the remaining 3 % (6/209) felt this should be done only in specific situations, e.g. when there is need to see a doctor, or if risk factors such as a positive family history for hypertension or metabolic syndrome are present. In 56 % (30/54) of the participating countries, obese children were currently screened for hypertension; in 20 % (11/54) of the countries contradictory answers within the country were given regarding whether or not obese children were screened for hypertension. According to 86 % (131/152) of the respondents who indicated that children are screened for hypertension in their country, screening was done by a paediatrician, in 32 % (49/152) by a general practitioner, and in 32 % (49/152) by preventive child health care (school nurse or physician) (multiple answers possible). Table 1 shows who performs the screening per continent.

**Table 1:** Screening for hypertension and preferred options for treatment of hypertension in obese children, per continent

	Total	Europe	Asia	N. America	S. America	Oceania
Screening done by						
Paediatrician	133 (57)	97 (55)	23 (70)	4 (40)	6 (75)	3 (50)
General practitioner	49 (21)	36 (21)	5 (15)	3 (30)	2 (25)	3 (50)
Preventive healthcare	50 (22)	42 (24)	5 (15)	3 (30)	–	–
Preferred treatment						
Lifestyle program	112 (57)	78 (58)	22 (55)	2 (40)	5 (63)	2 (40)
Medication	4 (2)	3 (2)	1 (3)	–	–	–
Both	79 (41)	54 (40)	17 (43)	3 (60)	3 (38)	3 (60)
Start medication						
After 12 months	10 (9)	10 (13)	–	–	–	–
After 6 months	69 (63)	48 (63)	14 (64)	1 (50)	3 (60)	3 (60)
After 3 months	13 (12)	8 (11)	3 (14)	1 (50)	–	1 (20)
After 1 months	3 (3)	–	2 (9)		1 (20)	–
Depends on patient	13 (12)	10 (13)	3 (14)		1 (20)	1 (20)
Preferred medication						
Diuretics	2 (1)	2 (20	–	–	–	–
Beta-blocker	5 (3)	4 (3)	1 (3)	–	–	–
Calcium antagonist	45 (23)	27 (21)	16 (40)	–	–	3 (43)
ACE-I/ARB	138 (73)	98 (74)	22 (55)	5 (100)	7 (100)	4 (57)

*ACE-I* angiotensin-converting enzyme (*ACE*)-inhibitors, *ARB* angiotensin receptor blockers

**Fig. 1:**
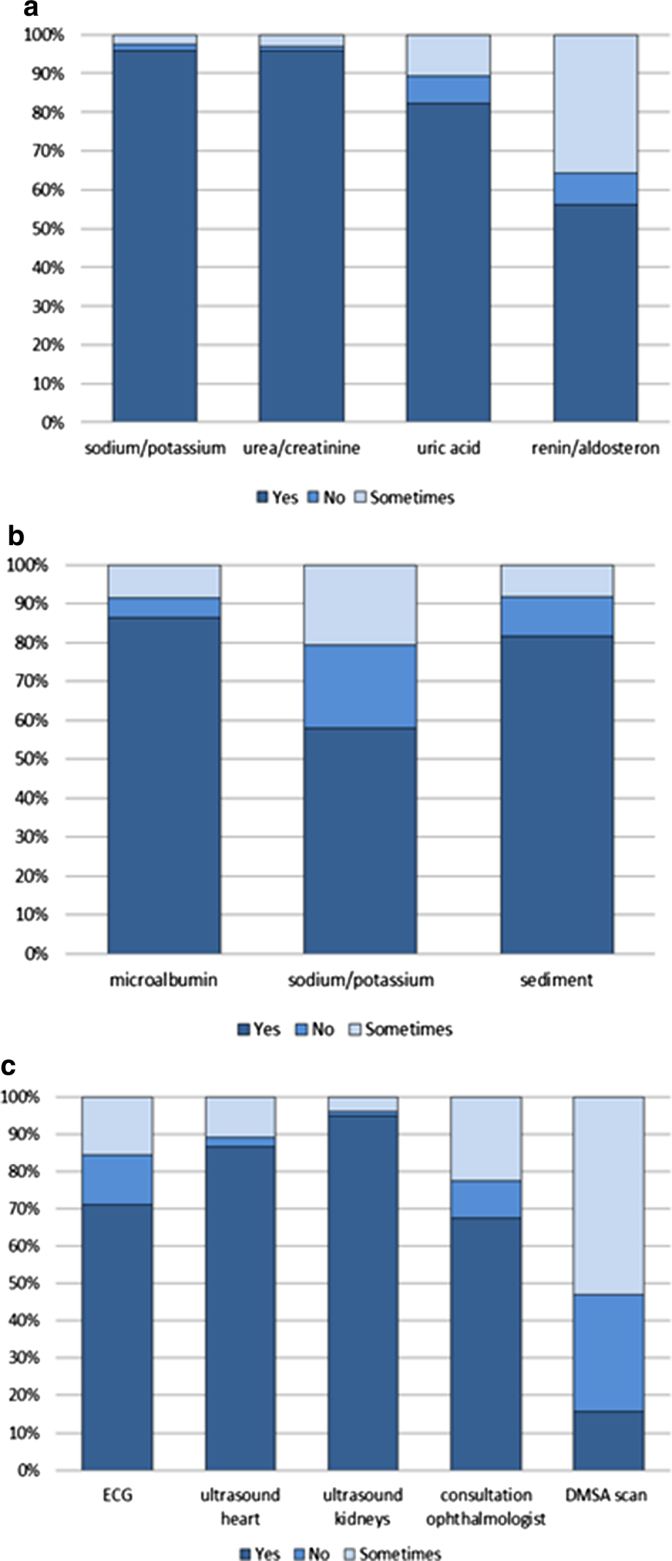
Use of diagnostic tests by paediatric nephrologists (n = 241) in obese children with hypertension to rule out other (secondary) causes of hypertension. **a** Diagnostic blood tests. **b** Diagnostic urine tests. **c** Other diagnostic tests. *ECG* electrocardiogram, *DMSA* *scan* dimercaptosuccinic acid renal scan

According to 88 % (174/197) of paediatric nephrologists, diagnosis of hypertension in obese children is performed by means of 24-h ambulatory blood pressure measurement (ABPM). There was no significant difference in use of ABPM to diagnose hypertension between respondents in Europe and Asia. If hypertension is diagnosed in obese children, several tests can be done to rule out other (secondary) causes of hypertension. Figure 1a–c display which tests were performed by the respondents. In Asia, significantly more paediatric nephrologist (92 %) refer obese children with hypertension to an ophthalmologist to check for hypertensive retinopathy in comparison to paediatric nephrologists in Europe (63 %) (p = 0.003). In Europe, tests for both plasma renin/aldosterone (p = 0.003) and urinary sodium/potassium (p = 0.02) are more frequently performed (respectively 64 and 66 %) than in Asia (respectively 43 and 42 %).

When hypertension is diagnosed in obese children, 45 % (88/195) of the respondents preferred to start treatment with a lifestyle program, 2 % (4/195) started with antihypertensive medication, and 40 % (78/195) with both. In addition, several paediatric nephrologists mentioned that the treatment of choice depends on the stage and severity of the hypertension and the presence of target organ damage. If a lifestyle program is preferred but hypertension persisted, 9 % (10/113) would start antihypertensive treatment after 12 months, 60 % (68/113) after 6 months, 13 % (15/113) after 3 months, 3 % (3/113) after 1 month, and 12 % (14/113) answered that it depends on the severity of the hypertension, and the presence of symptoms and target organ damage. There was no significant difference between Europe and Asia. Significantly more paediatric nephrologists in Europe (78 %) preferred angiotensin-converting enzyme (ACE)-inhibitors or angiotensin receptor blockers as the drug of first choice in comparison to paediatric nephrologists in Asia (58 %) (p = 0.01). 42 % of paediatric nephrologists in Asia preferred calcium antagonist as opposed to 22 % of European paediatric nephrologists. The use of beta-blockers and diuretics among the respondents was negligible. Table 1 shows the preferred options for treatment of hypertension in obese children per continent.

The respondents experienced several obstacles regarding screening, diagnosis and treatment of hypertension in obese children. A frequently mentioned bottleneck was the non-compliance of parents and children to treatment. Respondents stated that parents and children often have a lack of knowledge concerning obesity and hypertension, and a reluctant attitude towards its treatment and care. Another frequently mentioned bottleneck was the lack of necessary equipment, e.g. an ABPM, lack of appropriate blood pressure reference tables, and of appropriate size cuffs for primary screening by health care professionals. In addition, poor screening structures were reported as an obstacle: lack of infrastructure for the screening for hypertension in referrals and a lack of personnel. In addition, scarce and low-quality treatment programmes for obesity in children were mentioned. The frequency of the mentioned obstacles per continent is shown in Table 2.

**Table 2:** Most important obstacles experienced by paediatric nephrologists regarding screening, diagnosis and treatment of hypertension in obese children, per continent

	Total	Europe	Asia	N. America	S. America	Oceania
Non-compliance	78 (35)	41 (28)	27 (55)	4 (50)	5 (50)	1 (14)
Poor BP screening structures	36 (16)	21 (14)	9 (18)	3 (38)	1 (10)	2 (29)
Inadequate obesity treatment programmes	23 (10)	20 (14)	1 (2)	–	–	2 (29)
Lack of clear reference values and cuff sizes	18 (8)	12 (8)	5 (10)	–	1 (10)	–
Lack of ABPM	12 (5)	9 (6)	3 (6)	–	–	–
Lack of awareness of the problem as a healthcare issue	12 (5)	10 (7)	1 (2)	–	–	1 (14)
Poor diagnosis and treatment structures for hypertension	41 (19)	33 (23)	3 (6)	1 (13)	3 (30)	1 (14)
Total	220 (100)	146 (100)	49 (100)	8 (100)	10 (100)	7 (100)

*BP* blood pressure, *ABPM* ambulatory blood pressure monitoring

The respondents provided several suggestions for improving the screening, diagnosis and treatment of hypertension in obese children: (1) to establish an adequate system for screening for hypertension in all obese children, e.g. at school; (2) to raise national awareness of obesity and hypertension as a serious public health problem; (3) to establish educational programs for physicians, especially family doctors, on the principles of hypertension in obese children; (4) to create international guidelines on the screening, diagnosis and treatment of hypertension specific for obese children, including international reference values for blood pressure; (5) to make available equipment, such as an ABPM and several cuff sizes for primary care, and finances necessary for the implementation of screening; (6) that the treatment and counselling of obese children (with hypertension) be carried out by a multidisciplinary team including nutritionists, psychologists, physical education teachers, and specialized nurses; (7) that supervision of the treatment of obese hypertensive children should, however, be done by paediatricians or paediatric nephrologists and not by general practitioners.

## Discussion

Nearly all paediatric nephrologists were convinced that obese children should be screened for hypertension and nearly all regarded ABPM as the preferential diagnostic tool for confirming the diagnosis of hypertension. The respondents generally agreed on the need to perform a series of diagnostic tests to rule out secondary causes of hypertension and to look for consequences of hypertension (e.g. renal function, urine microalbumin, and ultrasound of heart and kidneys). However, plasma renin/aldosterone and a dimercaptosuccinic acid (DMSA) renal scan were regarded as unnecessary in obese children by half of the paediatric nephrologists. The majority of respondents (60 %) said they would aim to start antihypertensive medication within 6 months if lifestyle intervention was unsuccessful in reducing the blood pressure.

In a survey conducted among paediatric nephrologists in North America in 2005, ABPM was used by 63 % of respondents to diagnose or monitor primary hypertension in children [12]. In our study, ABPM was used by 88 % of the respondents; hence, in the last decade the use of ABPM seems to have increased. However, according to the European Society of Hypertension and the Fourth Report, ABPM should be used in selected cases and not as the golden standard for diagnosing hypertension in children [8, 9]. Existing normative values are based on relatively small groups of children, and there is need for expansion of reference values for paediatric ABPM. Although ABPM could certainly provide important information, interpretation of ABPM readings should be handled cautiously [8, 9, 13].

A possible consequence of hypertension in children is left ventricular hypertrophy. Almost 90 % of paediatric nephrologists in our sample perform an ultrasound of the heart to identify left ventricular hypertrophy in hypertensive obese children, as recommended by the European Society of Hypertension and the Fourth Report for all children with hypertension, not specifically for obese children with hypertension [8, 9]. A less common consequence of childhood hypertension is hypertensive retinopathy. In Asia, obese children with hypertension are significantly more often referred to an ophthalmologist to check for hypertensive retinopathy than in Europe. Studies on the effect of elevated blood pressure on retinal vasculature in children are limited and results are contradictory [14, 15]. To the best of our knowledge, there are no studies on the presence of retinal vascular abnormalities specifically in obese hypertensive children.

The most frequent causes of secondary hypertension in children are of renal origin [9, 16]. Almost all paediatric nephrologists in our study determine plasma creatinine and perform an ultrasound of the kidneys. This is in accordance with the recommendations of the European Society of Hypertension and the Fourth Report for all children with hypertension, not specifically for obese children with hypertension [8, 9]. Another point to highlight is that only 60 % of paediatric nephrologists in Europe and 40 % of paediatric nephrologists in Asia measure the plasma concentration of electrolytes in order to detect secondary forms of hypertension (e.g. hyperaldosteronism, although rare in children), even though this is recommended by the guidelines [8, 9].

Of the respondents, 42 % indicated that treatment should start with antihypertensive medication, and the majority of these stated it should be alongside a lifestyle programme. However, the European Society of Hypertension and the Fourth Report recommend that pharmacological treatment should be used in selected cases and only if a lifestyle programme has not accomplished enough change in blood pressure [9]. There was a significant difference in drug of first choice between Europe and Asia: in Europe ACE-inhibitors or angiotensin receptor blockers were significantly more often indicated as the drug of first choice than in Asia. ACE-inhibitors are recommended as the drug of first choice in obese hypertensive adults [17], and in children with primary hypertension [12]. Limited data are available on pharmacological treatment of hypertension specific for obese children.

Paediatric nephrologists perceived as an important obstacle the lack of consensus and guidelines for the diagnosis and treatment of hypertension specific for obese children. A lack of consensus regarding evaluation and management of hypertensive children has previously been recognized [12]. Uniformity in diagnosis and treatment of hypertension is of great importance in order to reliably determine the presence of comorbidity of obesity, and to be able to examine the effectiveness of treatment programs.

The most frequently mentioned suggestion for improvement of the current situation was to establish an adequate protocol for screening for hypertension in all obese children, e.g. at school. Yet, if a system of screening for hypertension is established there should also be opportunities for the diagnosis and treatment of obese children with hypertension.

A topic debated in the recent literature [1, 18–20] concerns the issue whether or not there should be separate reference values for overweight and obese children versus normal weight children. The reference values provided by the Fourth Report are based on both non-overweight and overweight or obese children [21]. Schwandt et al. argue that since overweight and obese children have substantially higher blood pressure values, separate blood pressure percentiles should be used for them. This would allow overweight and obese children to have a higher normal blood pressure than non-overweight children [1]. However, as Urbina and Falkner argue, an increased blood pressure in children imposes an increased risk for target organ damage and cardiovascular events [18]. Since the same is true for overweight and obese children, they should not intentionally be exposed to higher risks for adverse outcomes of elevated blood pressure. It could be argued that reference values should be based on normal weight children alone. Using reference values based on both normal weight and overweight/obese children combined leads to the risk that normal weight children with an elevated blood pressure may not be identified, since overweight and obese children raise the cut-off values [19].

A limitation of this study is the low response rate (10 %). The 214 respondents who completed questionnaires provided interesting information about current practice and expressed the need for improvements. However, these results may not be generalizable. Unfortunately, we do not have information about the non-respondents. It would have been interesting to compare characteristics of respondents versus non-respondents, such as type of practice (academic vs. private), region (country, and small town vs. large city). It is possible, therefore, that a selection bias has occurred. Another limitation is the small number of respondents per continent. Comparative analyses between the continents could not be performed due to the small numbers within the groups. Therefore, only Europe and Asia were compared. However, it is not a surprise that most continents were not well represented since the questionnaire was sent to members of the European Society for Paediatric Nephrology, and only European respondents were expected.

A limitation of the questionnaire is that it did not address what criteria are used by the respondents to diagnose hypertension (e.g. the definitions provided by the Fourth Report or local national blood pressure percentiles) or what criteria are used to diagnose obesity in children (e.g. body mass index percentiles provided by the US Center for Disease Control and Prevention, or the World Health Organisation, or local national body mass index percentiles, or the classification of the International Obesity Task Force).

A strength of the study is the broad variety of countries that participated, 54 countries in total, which provided a clear view of the current practice in different countries and cultures and what clinical care gaps are experienced. Although respondents from all countries indicated the need for an international guideline, it is questionable if having worldwide consensus on screening, diagnosis and treatment of hypertension in obese children, and thereby creating one universal guideline, is possible. Because of major differences between different parts of the world in terms of socioeconomics, genetics, etc., one international guideline might not be useful, and it would probably be preferable to create a basic model for a guideline and then adapt it for different parts of the world.

## Conclusion

The findings of this study emphasize the urgent importance of having clear recommendations and an international guideline for the screening, diagnosis and treatment of hypertension in obese children, since hypertension in obese children may need a different diagnostic and treatment approach from that suitable for children with secondary hypertension.

## Electronic supplementary material

Below is the link to the electronic supplementary material.

Supplementary material 1 (PDF 90 kb)

Supplementary material 2 (PDF 21 kb)
