# Supplementary material for: Screening, diagnosis and treatment of hypertension in obese children: an international policy comparison
Source: J Nephrol. 2016 Mar 3;30(1):119–25. doi: 10.1007/s40620-016-0277-6 (PMC5316390; doi:10.1007/s40620-016-0277-6)
Supplement: Supplementary file 1 — Supplementary material 1 (PDF 90 kb) [file 40620_2016_277_MOESM1_ESM.pdf]

Title: Screening, diagnosis and treatment of hypertension in obese children: an international policy comparison
Journal: Journal of Nephrology
Authors: Aleid JG Wirix, Jelle Verheul, Jaap W Groothoff, Jeroen Nauta, Mai JM Chinapaw, Joana E Kist-van Holthe
Address corresponding author: Aleid Wirix: Department of Public and Occupational Health, EMGO Institute for Health and Care Research, VU University Medical Center, van der Boechorststraat 7, 1081BT Amsterdam, the Netherlands. E-mail: a.wirix@vumc.nl, +3120-4445931

Supplementary Information 1. Questionnaire regarding screening, diagnosis and treatment of hypertension in obese children

Dear Colleague,

Paediatricians are increasingly confronted with childhood obesity. The Dutch Paediatric Association is currently developing a guideline for cardiovascular risk management and treatment of hypertension in obese children in integrated care with preventive child health care, general practitioners and paediatricians. We are very much interested how the care for obese children with hypertension in your country is organized.

Completing the questionnaire will not take more than 5-10 minutes of your time. The questionnaire is anonymous.

If you wish to receive the results of the study please enter your

e-mail address here: ……………………………@……………………….

General:

1. In which country do you live? …………………………………………..

2. You are a:

O Paediatrician
O Paediatric endocrinologist
O Paediatric cardiologist
O Paediatric nephrologist
O Other ……………………………………………………………….

3. You work at a (multiple answers possible):

O General hospital
O University hospital
O Private Clinic
O Outpatient clinic for obesity
O A special obesity clinic

Screening, diagnosis and treatment of hypertension in obese children:

4. In your country are obese children screened for hypertension?

O Yes
O No *(you will be directed to question no. 6)*

5. Obese children are screened by:

- O preventive child health care
  - O (school)nurse
  - O physician
- O general practitioner
- O paediatrician

6. In your opinion should blood pressure be measured in obese children?

O Yes O No O Sometimes, when…………………………….

7. Do you see obese children with hypertension?

O Yes
O No *(you will be directed to the end of the questionnaire)*

8. How do you confirm the diagnosis of hypertension in obese children?

O Measurement of blood pressure at the outpatient clinic
O Measurement of blood pressure during a hospital (day) admission
O 24-hour ambulatory blood pressure measurement
O Other:……………………………………………………………………….

9. Which diagnostic tests do you perform with obese children with hypertension to rule out other (secondary) causes of hypertension?

	Yes	No	Sometimes
Blood pressure arms/legs	O	O	O
Blood tests:			
sodium / potassium	O	O	O
uric acid	O	O	O
urea / creatinine	O	O	O
renin /aldosterone	O	O	O
Other blood tests:…………………………………………………………………….			
Urine tests:			
microalbumin	O	O	O
creatinine	O	O	O
sodium / potassium	O	O	O
sediment	O	O	O
Other urine tests:……………………………………………………………………			
Other tests:			
ECG	O	O	O
ultrasound of the heart	O	O	O

ultrasound of the kidneys	O	O	O
consultation with ophthalmologist	O	O	O
DMSA scan	O	O	O

Other tests:………………………………………………………………………………

10. Which treatment do you start for obese children with hypertension?

O Lifestyle program for obese children
O Antihypertensive medication *(you will be directed to question no. 12)*
O Both
O Other: ……………………………………………………………………………

11. If you started a lifestyle program for obese children but hypertension persists, after how long will you start antihypertensive medication?

O Not applicable
O I do not start antihypertensive medication
O After 6 months
O After 12 months
O After:……………………………………………………………………………..

12. Which antihypertensive medication is your drug of first choice

O diuretic
O beta-blocker
O calcium antagonist
O ACE inhibitor / Angiotensin receptor blocker (ARB)
O Other:……………………………………………………………………………..

13. Who performs follow-up for obese children with hypertension? *Multiple answers are possible*

O General practitioner
O Paediatrician
O Paediatric nephrologist
O Paediatric endocrinologist
O Paediatric cardiologist
O Other…………………………………………………………………………...

Bottlenecks:

14. Which bottlenecks do you experience regarding screening, diagnosis and treatment of hypertension in obese children?

1. ………………………………………………………………………………………..

2. ………………………………………………………………………………………..

3. ………………………………………………………………………………………..

4. ………………………………………………………………………………………………..

15. What would you suggest to improve the screening, diagnosis and treatment of hypertension in obese children?

1. ………………………………………………………………………………………………..

2. ………………………………………………………………………………………………..

3. ………………………………………………………………………………………………..

End of questionnaire

THANK YOU FOR FILLING OUT THE QUESTIONNAIRE!
