# Supplementary material for: Screening, diagnosis and treatment of hypertension in obese children: an international policy comparison
Source: J Nephrol. 2016 Mar 3;30(1):119–25. doi: 10.1007/s40620-016-0277-6 (PMC5316390; doi:10.1007/s40620-016-0277-6)
Supplement: Supplementary file 2 — Supplementary material 2 (PDF 21 kb) [file 40620_2016_277_MOESM2_ESM.pdf]

Title: Screening, diagnosis and treatment of hypertension in obese children: an international policy comparison
Journal: Journal of Nephrology
Authors: Aleid JG Wirix, Jelle Verheul, Jaap W Groothoff, Jeroen Nauta, Mai JM Chinapaw, Joana E Kist-van Holthe
Address corresponding author: Aleid Wirix: Department of Public and Occupational Health, EMGO Institute for Health and Care Research, VU University Medical Center, van der Boechorststraat 7, 1081BT Amsterdam, the Netherlands. E-mail: a.wirix@vumc.nl, +3120-4445931

Supplementary Information 2. Number of questionnaire respondents per country.

**Europe**	**136**	**Asia**	**53**
UK	17	Turkey	27
France	16	China	2
Portugal	15	Iran	2
Germany	11	Sri Lanka	2
Spain	10	Bangladesh	1
Russia	9	Israel	1
Italy	11	Japan	1
Serbia	8	Jordan	1
Poland	7	Kuwait	1
Croatia	6	Myanmar	1
Belgium	5	Pakistan	1
Greece	4	Philippines	1
Slovenia	4	Saudi Arabia	1
Latvia	3	South Korea	1
Czech Republic	2	Thailand	1
Denmark	2	**South America**	**9**
Finland	2	Argentina	4
Netherlands	2	Brazil	2
Sweden	2	Colombia	2
Albania	1	Chile	1
Bulgaria	1	**Oceania**	**7**
Georgia	1	Australia	6
Hungary	1	New Zealand	1
Lithuania	1	**North America**	**5**
Moldova	1	United States	3
Norway	1	Canada	1
Slovakia	1	Haiti	1
Switzerland	1	**Africa**	**0**
